# Disseminated *Fusarium* infection in a patient with acute lymphoblastic leukemia: A case report and review of the literature

**DOI:** 10.3892/ol.2013.1738

**Published:** 2013-12-06

**Authors:** YI-SHENG LIU, NING-CHI WANG, REN-HUA YE, WEI-YAO KAO

**Affiliations:** 1 Division of Hematology and Oncology, Department of Medicine, Tri-Service General Hospital, National Defense Medical Center, Taipei 114, Taiwan, R.O.C.; 2 Department of Medicine, Taichung Armed Forces General Hospital, Taichung 41152, Taiwan, R.O.C.; 3 Division of Infectious Disease, Department of Medicine, Tri-Service General Hospital, National Defense Medical Center, Taipei 114, Taiwan, R.O.C.; 4 Division of Hematology and Oncology, Department of Medicine, Taipei Tzu Chi General Hospital, Taipei 23142, Taiwan, R.O.C.

**Keywords:** *Fusarium*, *Aspergillus*, acute leukemia, neutropenia

## Abstract

*Fusarium* is a common soil mold. In severely immunocompromised patients, this fungus may cause disseminated disease and is often confused with *Aspergillus*, as the two pathogens have similar histopathological appearances. Disseminated *Fusarium* infection may cause significant morbidity and mortality in immunocompromised patients. The current case report presents a 20-year-old male with acute lymphoblastic leukemia who developed disseminated *Fusarium* infection during induction chemotherapy. Early diagnosis and treatment is extremely important since the mortality rate is extremely high in such patients. The clinician must consider that the clinical presentation of *Fusarium* infection resembles that of *Aspergillus*. There is no optimal treatment for patients with *Fusarium* infection; however, combination antifungal therapy may have benefit without significant toxicity.

## Introduction

Invasive fungal infections are leading causes of mortality and morbidity in patients with hematological malignancies and prolonged neutropenia following chemotherapy (1,2). In general, fluconazole is used as a prophylactic antifungal treatment for patients with hematological malignancies who are predicted to exhibit prolonged neutropenia following aggressive chemotherapy. Thus, mold infections become more common in patients with fluconazole prophylaxis and *Fusarium* is the second most common cause of mold infection. The incidence of *Fusarium spp.* infections in patients with acute leukemia in Europe is 0.06% (3) and was 1.2% among 750 allogeneic and 0.2% among 1,537 autologous marrow transplant recipients in the United States (4). Disseminated fusariosis accounts for 70% of *Fusarium* infections in immunocompromised patients, particularly in patients with acute leukemia with prolonged and profound neutropenia, and patients undergoing hematopoietic stem cell transplantation (5).

## Case report

**Figure 1:**
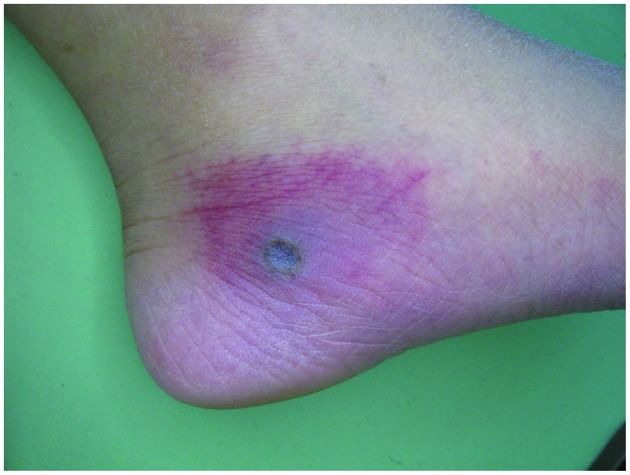
Classical single lesion with indurated erythematous papule and central necrosis.

**Figure 2:**
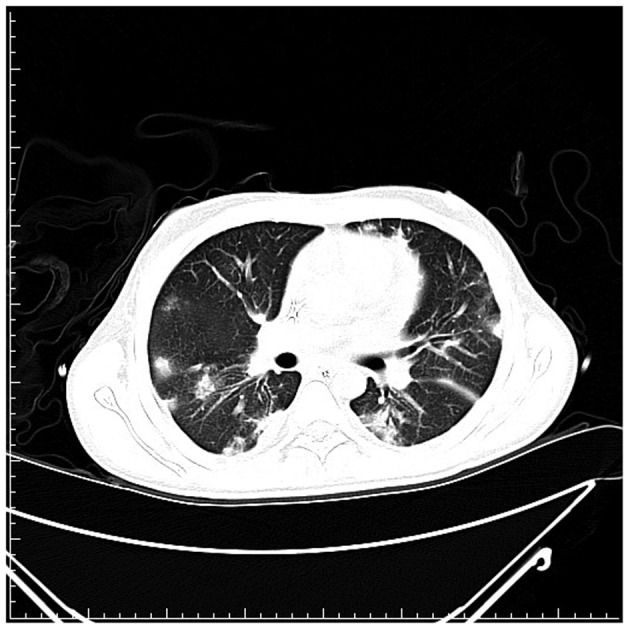
Radiological observations may indicate non-specific infiltrates to nodular and/or cavity formation.

**Figure 3:**
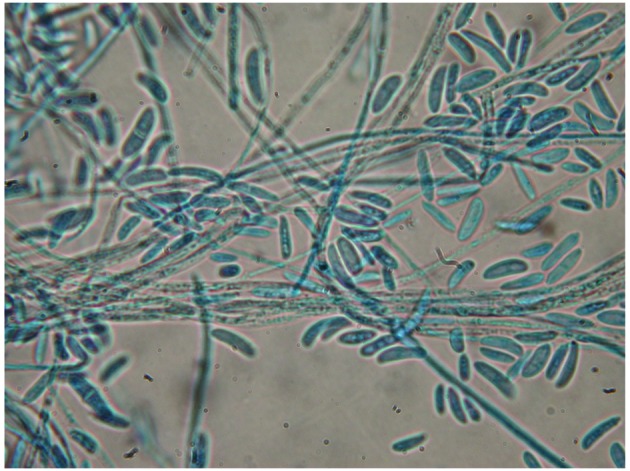
Microscopically, *Fusarium* filaments were hyaline, septate and 3- to 8-μm in diameter, typically branching at acute or right angles.

A 20-year-old male was diagnosed with acute lymphoblastic leukemia, precursor B cell type and received induction chemotherapy with TPOG-ALL-2002 VHR protocol (6) with partial remission in the induction course. The patient did not finish the consolidation course due to prolonged neutropenia. Later, the patient relapsed and received chemotherapy with FLAG-IDA (idarubicin, fludarabine, cytarabine and G-CSF) (7). The patient exhibited prolonged febrile neutropenia for more than one month during the initial course of chemotherapy, but exhibited rapid relapse again, soon following neutropenia recovery. In the second course of treatment, the patient received 400 mg oral fluconazole daily as an antifungal prophylaxis. The febrile neutropenia was found two days later. Antibiotic treatment with imipenem/cilastatin (500 mg, i.v., every 6 h), vancomycin (1,000 mg, i.v., every 12 h) and micafungin (100 mg, i.v., daily) was then administrated. After four days, multiple skin lesions, starting from the legs and spreading to the face and upper extremities were identified. The lesions exhibited necrotic centers surrounded by spreading erythema (Fig. 1). The lesions worsened and the antifungal treatment was replaced with caspofungin (100 mg, i.v., daily). A biopsy of the skin lesions showed the presence of hyphae occupying the vascular space. The Gomori methanamine silver and periodic acid-Schiff stains were positive. The histopathological diagnosis was angioinvasive aspergillosis and treatment with voriconazole (200 mg, i.v., every 12 h) was initiated in addition to caspofungin with poor response. The amphotericin B was not administered due to ethical issues (the patient and family refused further aggressive treatment owing to refractory acute lymphoblastic leukemia with poor therapeutic response). A high-resolution computed tomography scan of the lungs found multiple speculated and round consolidative densities in the lungs (Fig. 2). After three weeks, the sputum and skin tissue cultures finally demonstrated *Fusarium spp.* colonization (Fig. 3). The patient succumbed to the disease two months later. Written consent was provided from the patient’s family.

## Discussion

*Fusarium* is widely distributed in soil, plants and air and is common in tropical and temperate regions. This pathogen can cause a broad spectrum of human diseases, which may be locally invasive or disseminated. In immunocompromised patients, particularly in high-risk patients (those with prolonged and profound neutropenia), *Fusarium* infection is typically invasive and disseminated (5). In a previous study of invasive fusariosis in 84 patients with hematological malignancies, patients with acute leukemia occurred most frequently (56%) and the majority of patients (83%) were neutropenic at diagnosis (8). The incidence of *Fusarium spp.* infection in patients with acute leukemia in Europe is 0.06% (3). Previously, in a 10-year follow-up study from a US institution, the incidence of *Fusarium* infection was 1.2% among 750 allogeneic and 0.2% among 1,537 autologous marrow transplant recipients (4).

*Fusarium* infection in immunocompromised patients is airborne or inoculated through the breakdown of the skin barrier. Skin lesions may involve any skin sites, with predominance in the extremities. *Fusarium* infection presents more frequently as metastatic skin lesions with initial presentation of subcutaneous lesions, to erythematous indurations, followed by target-like central necrotic lesions (4). The lesions are evoked rapidly within one to five days, at various stages of evolution and occasionally with myalgias (9). Skin biopsies are extremely easy to perform and confirm clinical suspicion. However, the histopathological images of skin lesions resemble those of *Aspergillus* infections. The two infections exhibit vascular invasion and branching septate hyphae (4); therefore, identification from *Aspergillus* may be difficult.

As with the case of *Aspergillus*, the radiological observations of pulmonary *Fusarium* infection are non-specific, including non-specific infiltrates to nodular and/or cavity formation, depending on the timing of the examination (9). The prognosis is considerably worse in patient pulmonary infiltrates (10).

The definite diagnosis of *Fusarium* infection requires *Fusarium spp.* isolation from blood and tissue culture (such as the skin, lungs and sinuses). However, the *Fusarium* species may contaminate laboratory specimens and pseudo-outbreaks of fusariosis may occur (11). Thus, the clinician must be cautious of interpretation. Unlike *Aspergillus*, the *Fusarium* species may be isolated from cultures of blood samples in 40% of cases. With the presence of disseminated skin lesions, the presence of the fungus in blood cultures may increase to 56% (8). In the majority of cases, fungemia is likely to develop in a median of five days (range, one to 10 days) following the appearance of skin lesions (9). However, in the present case, the blood culture did not yield *Fusarium* species despite disseminated skin lesions.

For patients with persistently febrile neutropenia, empiric treatment with caspofungin is initially recommended and liposomal amphotericin B is an alternative choice (12). There is no treatment guideline for *Fusarium* infection. Nucci *et al*(8) suggested high-dose amphotericin B or lipid formation of amphotericin B since specific *Fusarium* species may be resistant to azoles. The greatest challenge to clinicians is the early diagnosis of the *Fusarium* infection and preemptive treatment, since the histopathology of *Fusarium* may be confused with *Aspergillus* and the galactomannan test may not be useful for distinguishing *Fusarium* from *Aspergillus*(13). Despite aggressive treatment, the survival rate of the *Fusarium* infection in patients with persistent neutropenia is only 4% (8). Early granulocyte recovery is significantly associated with favorable response and survival and growth factors (G-CSF or GM-CSF) or granulocyte transfusions, which may shorten the neutropenic period to aid immunity recovery (15). Combination antifungal therapy is well-tolerated with acceptable minor toxicity (14,15) and, theoretically, may have benefits to stabilizing the infection and preventing fatal progression. However, such data are limited in hematological patients with *Fusarium* infection. In a previous report of immunocompromised patients with disseminated *Fusarium* infection, 70% of patients (14 cases) exhibited a positive response to combination antifungal therapy (16). In one multicenter retrospective study of 61 hematological patients with confirmed or predicted invasive mold infections, comparing liposomal amphotericin B plus caspofungin, liposomal amphotericin B plus a triazole and voriconazole plus one echinocandin drug, no statistical differences were identified among these groups at the end of treatment and 12-week survival (15). In the current case, the patient was recommended amphotericin B plus caspofungin or amphotericin B plus voriconazole treatment in combination; however, the patient and family refused due to ethical issues. The optimal treatment for patients with *Fusarium* infection requires further investigation and well-designed clinical trails to address this issue.
